# Modified Phospholipid Vesicular Gel for Transdermal Drug Delivery: The Influence of Glycerin and/or Ethanol on Their Lipid Bilayer Fluidity and Penetration Characteristics

**DOI:** 10.3390/gels11050358

**Published:** 2025-05-13

**Authors:** Marwa H. Abdallah, Mona M. Shahien, Hemat El-Sayed El-Horany, Enas Haridy Ahmed

**Affiliations:** 1 Department of Pharmaceutics, College of Pharmacy, University of Ha’il, Ha’il 81442, Saudi Arabia; 2 Department of Pediatrics, College of Medicine, University of Ha’il, Ha’il 81422, Saudi Arabia; m.shahin@uoh.edu.sa; 3 Department of Biochemistry, College of Medicine, University of Ha’il, Ha’il 81422, Saudi Arabia; h.elhorany@uoh.edu.sa; 4 Department of Anatomy, College of Medicine, University of Ha’il, Ha’il 81442, Saudi Arabia; e.haridy@uoh.edu.sa; 5 Department of Anatomy and Embryology, Faculty of Medicine, Ain Shams University, Cairo 11566, Egypt

**Keywords:** ethosomes, glycerosomes, glycethosomes, gel, ethanol, glycerin, penetration, transdermal

## Abstract

This review explores the enhanced transdermal therapy of several skin disorders with the application of carriers comprising phospholipid vesicular gel systems. Topical drug delivery has several advantages compared to other administration methods, including enhanced patient compliance, the avoidance of the first-pass impact associated with oral administration, and the elimination of the need for repeated doses. Nonetheless, the skin barrier obstructs the penetration of drugs, hence affecting its therapeutic efficacy. Carriers with phospholipid soft vesicles comprise a novel strategy used to augment drug delivery into the skin and boost therapeutic efficacy. These vesicles encompass chemicals that possess the ability to fluidize phospholipid bilayers, producing a pliable vesicle that facilitates penetration into the deeper layers of the skin. Phospholipid-based vesicular carriers have been extensively studied for improved drug delivery through dermal and transdermal pathways. Traditional liposomes are limited to the stratum corneum of the skin and do not penetrate the deeper layers. Ethosomes, glycerosomes, and glycethosomes are nanovesicular systems composed of ethanol, glycerol, or a combination of ethanol and glycerol, respectively. Their composition produce pliable vesicles by fluidizing the phospholipid bilayers, facilitating deeper penetration into the skin. This article examines the impact of ethanol and glycerol on phospholipid vesicles, and outlines their respective manufacturing techniques. Thus far, these discrepancies have not been analyzed comparatively. The review details several active compounds integrated into these nanovesicular gel systems and examined through in vitro, in vivo, or clinical human trials involving compositions with various active molecules for the treatment of various dermatological conditions.

## 1. Introduction

One of the least invasive and most patient-friendly methods of administering therapeutic agents is transdermal drug delivery. By concentrating drug molecules in a specific area of the skin, it can increase the bioavailability of drugs while reducing the likelihood of unanticipated side effects [1,2]. As a result, transdermal drug delivery is a desirable substitute for hypodermic injection in oral administration [3]. The breakdown of the skin barrier through delipidization, the hydration and disruption of lipid packing, and modifications in the drug/skin thermodynamic condition are the processes that improve transdermal penetration [4]. Over the past ten years, transdermal drug delivery systems have become increasingly important because of their many benefits, which include physicochemical protection for various drugs; better patient compliance; suitability for patients who are unconscious or vomiting; avoidance of first-pass metabolism, which increases the drug’s bioavailability; a lower risk of toxic side effects; and decreased frequency of dose administration [5,6,7,8]. The technique makes it possible to provide drugs without being constrained by the fluctuating pH, enzyme activity, and intestinal flora of the digestive environment. This not only protects the drug’s integrity but also lessens the difficulties of oral delivery, where enzymatic breakdown and gut pH might affect the stability and effectiveness of the drug [9]. In addition to its many benefits, the transdermal drug delivery system has many drawbacks. Large molecules such as proteins, which have well-adjusted lipophilicity and measurable solubility in both water and oil, can only be delivered with limited efficiency. Drug molecules with comparatively high pharmacological potency are also good candidates for this delivery technique [10,11].

**Figure 1:**
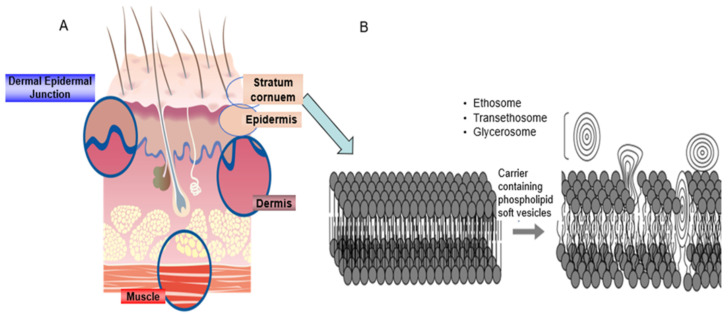
Schematic illustrations of (**A**) the skin structure; (**B**) the mechanism of action of carriers containing phospholipid vesicles [20].

The cellular structure of the skin means that there is a necessity for employing advanced delivery systems to surmount these permeability obstacles. The skin serves as the body’s largest organ, providing a crucial barrier against external substances. The stratum corneum (SC), the outermost layer of the skin, is composed of nonviable and keratinized cells. This layer serves as an efficient barrier, effectively retaining water within the body while preventing the entry of external compounds. The majority of therapeutic molecules demonstrate an inadequate capacity to effectively penetrate the skin barrier. The lipid molecules present in the stratum corneum, predominantly cholesterol, ceramides, and fatty acids, are crucial in defining the barrier function of this layer [12]. The lipids are organized into diminutive organelles, resulting in the formation of lamellar granules that merge in an edge-to-edge fashion, ultimately creating flattened lamellar disks arranged in paired bilayers [13]. The primary pathway for molecular penetration is the intracellular route traversing these lipid bilayers of the stratum corneum [14]. The viable epidermis and dermis consist of layered tissues characterized by a significant water concentration, which serves as a hydrophilic environment for molecules to navigate their path from the stratum corneum to the blood vessels. The utilization of chemical penetration enhancers, which are molecules that engage with skin constituents to facilitate the movement of active compounds, has long been recognized as an effective strategy for traversing the skin barrier layers and enabling the penetration of active molecules [15]. Over the past forty years, nanocarriers have been developed for drug delivery via the skin, with this method being utilized to treat a variety of illnesses. Solid lipid nanoparticles, nanoemulsions, and polymeric nanoparticles [16,17,18] are examples of nanocarriers. Around 40 years ago, phospholipid soft vesicular systems were developed as novel penetration enhancers for skin. These systems are identified by their fluid lipid bilayers, which are caused by the presence of compounds such as ethanol and glycols. The solvents in these carriers fluidize and break down the lipid bilayers in the SC when applied topically, allowing the soft vesicles to enter the skin at a greater deepness and release their active ingredients [19] (Figure 1). The topical delivery of drugs included in these novel carriers has drawn more interest as a means of treating skin diseases.

In this review, we present an extensive examination of transdermal drug administration using modified phospholipid vesicular gels, emphasizing both their structural and functional advantages over traditional vesicular systems. We go over the main formulation techniques, important characteristics, and underlying processes that lead to their enhanced drug retention and skin penetration. We also provide an overview of recent developments in the use of these systems for different therapeutic agents, highlighting their potential benefits in terms of patient compliance, stability, and regulated release. This study attempts to assist researchers and formulators in choosing the best vesicular gel system based on drug characteristics and intended therapeutic use by addressing formulation problems and translational considerations.

## 2. Methodology

In this review, we have provided a summary of the recent developments in the use of nanovesicles gelling systems containing ethanol and/or glycerin as a nanocarriers for the delivery of drugs primarily used to treat dermatological disorders. A comprehensive literature search was conducted using publication search engines like Science Direct, Elsevier, Scopus, and PubMed for relevant articles published in the past decade (2010–2024). Key terms including glycethosomes, glycerosomes, ethosomes, permeation enhancers, modified liposomes or niosomes, glycerin, and ethanol were employed to browse the literature.

To ensure the quality and relevance of the studies included, the following inclusion criteria were applied:

- Studies published in the English language.
- Research articles, reviews, and clinical studies that provided significant insights into the preparation, characterization, and application of nanovesicular systems for drug delivery, especially in the context of dermatological disorders.
- Studies that were published within the time frame of 2010–2024.

The exclusion criteria involved the following:

- Studies that did not specifically address the use of ethanol and/or glycerin-based nanovesicular systems.
- Articles not related to dermatological drug delivery or those focused on non-relevant therapeutic areas

To ensure the robustness of our findings, we prioritized research articles, reviews, and clinical studies that were published within this time frame and offered significant insights into the preparation, characterization, and application of these vesicular systems.

## 3. Phospholipid-Based Nanovesicles

Nanovesicles are particles made of amphoteric molecules, including phospholipids or surfactants, that surround an aqueous phase to form colloids of one or more concentric lipid bilayers [21]. Drugs that are hydrophilic or lipophilic can be encapsulated in vesicles in the hydrophilic core or lipidic bilayer, respectively [22]. They can reduce the risk of drug toxicity by delivering drugs to the intended action location. Moreover, it has been established that vesicular delivery techniques are better than traditional ones [23]. Since vesicles’ lipidic components may penetrate and alter the stratum corneum’s lipid matrix, it has been established that they can improve the skin penetration of drugs [24]. Additionally, vesicular systems enhance the properties of transdermal formulations in the nanoscale size range.

### 3.1. Classical Phospholipid-Based Nanovesicles

Liposomes are spherical vesicles that are made of one or more phospholipid bilayers. They are utilized extensively in drug delivery systems due to their good biocompatibility, capacity to encapsulate a wide range of medicines, and possibility for targeted delivery. Traditional liposomes have limited use due to their stability issues as a result of lipid degradation, the leakage of the encapsulated drug, and short shelf life, in addition to their rapid clearance from the bloodstream [25]. In light of this, several advanced liposomes have been manufactured by altering their composition [26]. These modifications make liposomes more stable and enable them to better traverse the barriers that exist within the body, which ultimately results in boosting penetration.

### 3.2. Modified Phospholipid-Based Nanovesicles Containing Glycerol and/or Ethanol for Transdermal Drug Administration

Touitou [27] initially proposed ethosomal phospholipid vesicles, which consist of phospholipids and ethanol (20–50% *w*/*w*), and optionally include water, glycols, and medicated compound. Short-chain alcohols like ethanol or isopropyl alcohol can be used in ethosomal vesicles, with the possibility of including glycols like propylene glycol and Transcutol^®^. Ethosomes are a kind of advanced nanocarrier that are based on lipids and are comparable to liposomes. Ethosomes are recognized as a novel drug delivery system that can significantly enhance drug penetration through the stratum corneum. However, they are distinguished by the presence of a high concentration of ethanol. Ethosomes are pliable vesicular structures mostly including phospholipids, water, and ethanol. Furthermore, the use of an effective penetration enhancer such as ethanol facilitates the formation of soft, flexible vesicles, enabling the enhanced permeation of vesicles through the skin’s layers. Consequently, ethosomes facilitate the transdermal release of the encapsulated drug [28,29]. It is recognized as a lamellar-shaped vesicular system with an elastic membrane that improves drug delivery and boosts its solubility, hence enhancing its encapsulation into the carrier [30]. Ethosomes have received a lot of attention because of their improved capacity to permeate the skin and consequent effective drug delivery. Ethosomes include high concentrations of low molecular weight alcohols such ethanol, propylene glycol, and isopropanol as penetration enhancers [31]. It was initially demonstrated by Touitou et al. that lipid vesicular systems are capable of coexisting with a high concentration of ethanol ranging from 20% to 45% *v*/*v* and forming multi-lamellar vesicles, which were subsequently referred to as ethosomes [32]. Ethanol has the ability to soften ethosome vesicles, facilitate drug distribution in the stratum corneum, and fluidize phospholipid bilayers. Ethosomes have a higher skin permeability than liposomes, which results in a significant increase in the amount of drugs that are able to be absorbed by the body membranes. According to variable studies [29,32], ethosomes have significant potential to enable the delivery of drugs to the deeper layers of the skin. Ethosomes interact more effectively with skin lipids than liposomes, hence enhancing the dispersion of active substances compared to liposomes [33]. The interaction of ethanol with the lipid molecules in the polar head group area reduces the transition temperature of the lipids in the stratum corneum. These facilitate the drug’s penetration into the deeper layers of the skin by enhancing fluidity and reducing lipid multilayer density. Moreover, ethanol enhances the smoothness and pliability of vesicles, promoting deeper infiltration into the epidermis [34,35].

**Figure 2:**
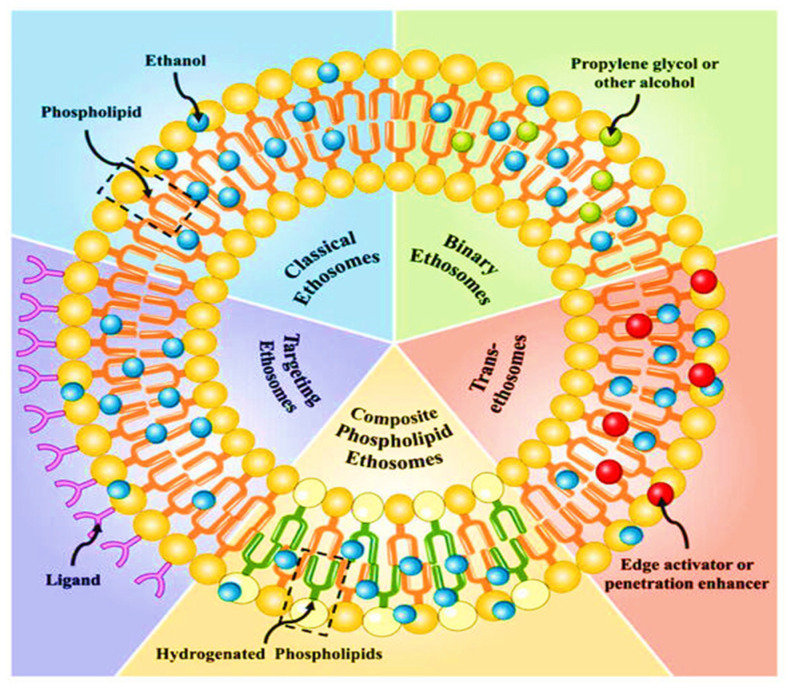
Different categories of ethosomes [39].

Ethosomes can be categorized based on their composition into several types. Each category exhibits unique characteristics and benefits (Figure 2). Conventional or classical ethosomes are stable drug delivery vehicles comprising phospholipids, cholesterol, ethanol, and water in different proportions. These vehicles facilitate the effective and efficient transportation of active ingredients [36]. Classical ethosomes have a higher ethanol content than conventional liposomes, leading to enhanced permeability. Zhang et al. synthesized ethosomal and liposomal vesicles containing psoralen. The transdermal flux and dermal accumulation of drug in ethosomal vesicles were recorded as 3.50 and 2.15 times greater than the values found with liposomal vesicles [37]. Yucel et al. created ethosomal and liposomal vesicles containing rosmarinic acid. This research demonstrated that ethosomal systems might improve drug penetration through human skin and exhibited a greater transdermal flux compared to liposomal vesicles [38]. The aforementioned data indicate that ethosomes have superior permeability relative to conventional liposomes.

Binary ethosomes are generated by augmenting conventional ethosomes through the use of a lower-chain alcohol (propylene glycol) and ethanol combination during the manufacturing procedure, rather than utilizing just ethanol. This lowers the quantity of ethanol and its volatility, enhancing drug solubility and formulation stability while facilitating drug penetration [36,40,41]. Numerous researchers have developed binary ethosomes to enhance formulation stability and augment skin penetration. Saadallah et al. developed tazarotene-incorporated binary ethosomes (TZ-BES). The optimized ethosomal formulation was combined with carbopol gel. In comparison to the tazarotene gel (43.54%), the optimized ethosomal gel demonstrates a significantly greater drug release (89.22%). After six hours, the permeability of the optimized ethosomal gel through rat abdomen skin was measured at 66.22%, significantly exceeding that of the control drug–gel, which was 24.67%. The flux of the optimized ethosomal gel was 2.68 times greater than that of the plain drug–gel. In conclusion, ethosomal gel presents a viable alternative for the topical administration of tazarotene [42]. Zhang et al. developed ethosomal, binary ethosomal, and transfersomal vesicles with terbinafine hydrochloride. This research demonstrated that binary ethosomal vesicles were the most efficacious in facilitating drug absorption through the skin in comparison to ethosomes and transfersomes [43].

Song et al. [19] were the first to demonstrate transethosomes, an advanced development of ethosomal systems.. The advantages of both ethosomes and transfersomes have been combined in this type of vesicle to create a single formulation [44,45]. Transethosomes are created by incorporating surfactant or penetration enhancer, such as sodium cholate, sodium deoxycholate, Tween 20, or Span 60, into traditional ethosomes [46]. The surfactant is integrated into the phospholipid bilayer, increasing the distance between phospholipid molecules, disturbing the bilayer’s structure, and making the ethosomes more fluid. The ethosomes distort and penetrate the stratum corneum when the skin is hydrated, enhancing the drug’s transdermal absorption.

Adnan et al. manufactured transethosomes of apigenin with Span 80 as the surfactant [47], which were optimized using Box–Behnken design. The ex vivo permeation of transethosomal gel was higher than the penetration of conventional gel after 24 h. A cytotoxicity investigation demonstrated that transethosomal gel markedly decreases cell viability in comparison to the standard formulation. Because of the prolonged release of the drug and increased permeability of apigenin through the skin, the results suggested that the topical use of apigenin transethosomal gel would be a better treatment strategy for skin cancer.

A study by Abdallah et al. developed erythromycin-loaded transethosomes which were optimized using a Box–Behnken design [48]. The optimum transethosomal vesicles was subsequently converted into a cinnamon oil-based emulgel utilizing hydroxylpropylemethyl cellulose as a gelling agent. The optimized transethosomal emulgel exhibited the greatest transdermal flux and demonstrated significantly superior antibacterial efficacy compared to conventional gel. The application of erythromycin was then enhanced with cinnamon oil, which ultimately had a significant impact on bacterial proliferation. Ultimately, these findings indicated that the transethosomal emulgel system serves as an alternate method for administering erythromycin in the treatment of cutaneous bacterial infections.

Glycerosomes are lipid-based vesicles that are comparable to liposomes but are characterized by a larger concentration of glycerol; they typically contain between 10 and 50 percent (volume/volume) glycerol [49]. Glycerosomes can substantially increase the stability of phospholipid vesicles by incorporating glycerol into typical liposomes [50]. For the first time, Manca et al. [50] discussed the possibility of using glycerosomes for the percutaneous administration of diclofenac. High glycerol concentration increases the fluidity of the phospholipid bilayers, which in turn improves stability, drug encapsulation, and permeability. Moreover, the increased viscosity of glycerosomes has a positive impact on their stability in comparison to liposomes [51,52]. Glycerol has the potential to operate as an edge activator for phospholipid bilayers. The use of glycerosomal systems has been investigated for the purpose of administering medicinal substances via several routes of administration, such as topically [53], transdermally, and intranasally [54]. Furthermore, there has been a new emphasis placed on the investigation of their capacity to transmit medicines by inhalation [55]. Therefore, in order to further improve the transdermal characteristics of the vesicles, the benefits that glycerosomes and ethosomes have to offer have been combined.

This novel vesicular system is a non-toxic and harmless topical drug delivery mechanism. Glycerosomes are not reliant on transition temperatures for their creation. They may be synthesized at an ambient temperature (30 or 25°), in contrast to traditional liposomes. They boost drug penetration in the stratum corneum and facilitate its delivery to the deeper layers of the skin by functioning as edge activators and penetration enhancers. These vesicles exhibit enhanced entrapment, fluidity, and stability. Stability is enhanced by altering the fluidity of the lipid bilayer and by creating viscous formulations [56]. Additionally, it creates malleable and stretchy vesicles. Glycerol, due to its viscous properties, uniformly distributes over the skin without any leakage of the active medicinal component, in contrast to traditional liposomes [51]. Glycerosomes have the ability to alter the arrangement of hydrophilic chains in phospholipids, hence influencing the interactions among other vesicles within the system. The dielectric constant of the system may vary in glycerosomes [57]. Glycerosomes enhance the flexibility of the epidermis, resulting in the enhanced hydration of the stratum corneum and reducing barriers to transdermal drug administration. These vesicles are distinctive in that they may function as both penetration-enhancing and elastic vesicles.

In conclusion, glycerosomes are novel vesicular structures comprising glycerol in diverse proportions. These devices can bypass skin barrier layers and enable the drug to reach deeper dermal regions, and are primarily utilized for topical drug administration owing to their capacity to enhance skin penetration, augment drug trapping in vesicles, and produce flexible, more fluidic vesicles. Glycerol functions as a modulator for the epidermal layers by altering their configuration. This technique has mostly been utilized for transdermal drug delivery; however, Manca et al. formulated glycerosomes of curcumin for pulmonary administration [58]. These vesicles could be readily produced at an ambient temperature and do not necessitate reaching the transition temperature of phospholipids. The entrapment of larger quantities of drugs can occasionally lead to delayed administration. These formulations have demonstrated significant stability, fluidity, entrapment efficiency, and viscosity during the past several years. These vesicles may be utilized for ocular drug delivery in the near future, since they will facilitate the administration of medicines with inadequate corneal penetration and low entrapment in liposomes.

**Figure 3:**
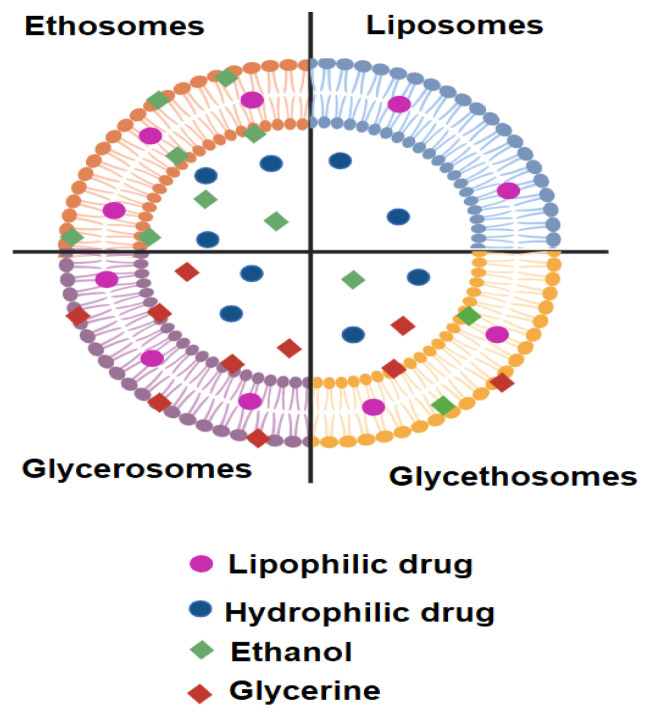
Schematic representation of various nanovesicles structure.

Glycethosomes are a specific type of phospholipid nanovesicles that are composed of ethanol and glycerin; see Figure 3. They are considered as a modified form of ethosomes and glycerosomes [59]. Glycethosomes are highly flexible and show a high degree of deformability, which lets them pass through intact mucosal membranes and skin effectively. They have the ability to improve the stability and bioavailability of drugs. Due to their capacity to enhance drug encapsulation efficiency and membrane penetration, they have demonstrated promising results in intranasal, transdermal, and oral drug delivery.

### 3.3. Phospholipid Vesicular Gel Systems

#### 3.3.1. Formulation of the Modified Types of Phospholipid Vesicular System Containing Ethanol and/or Glycerin

The literature reports that ethosomes are generally prepared using two primary methods: a cold method and a hot method. The most popular and extensively utilized technique for ethosomal preparation is the cold procedure. In this procedure, drugs, phospholipids, and other lipid components are dissolved in ethanol in closed containers at room temperature while being constantly stirred. If propylene glycol is present, it is then added. After that, water is gradually added to the produced mixture while being continuously mixed until ethosomal vesicles form. The final ethosomal dispersion is continuously stirred for thirty minutes at room temperature.

The other method is the hot approach, which involves the dissolving of the phospholipid mixture in water heated to 40 °C as described by Touitou et al. [32] and Chauhan et al. [35]. Next, the aqueous phase is mixed with the organic phase containing ethanol and optional propylene glycol. Depending on whether the medicine is hydrophilic or hydrophobic during production, it can be dissolved in either the aqueous phase or organic phase with ethanol. Since there is no solvent evaporation involved, these preparation techniques are eco-friendly [4]. The size of the ethosomal vesicles may be adjusted in both procedures using extrusion or sonication techniques.

Moreover, an advanced technique was used for Paeonol ethosomal preparation [60]. Soybean phospholipid, cholesterol, and pentol were dissolved in ethanol and mixed in the required ratios, followed by extraction. Ultrapure water was then added and the two phases were combined in a nucleic acid lipid nanoparticle preparation chip. The flow rate ratio between the aqueous and alcoholic phases was set at 3:1 (*v*/*v*) with total flow rates of 200–1000 μL/min. Microfluidic technology allows for the controlled mixing of phases (e.g., aqueous and alcoholic) at specific flow rates, which leads to the formation of well-defined vesicles (such as ethosomes). Microfluidics is valued in drug delivery systems because it enables uniform particle size, high encapsulation efficiency, and the ability to scale up or modify the process with precision [61].

Glycerosomes were manufactured by a thin film hydration approach, as demonstrated by Manca et al. and Alam et al. [56,62]. A phospholipid and cholesterol mixture was precisely measured and dissolved in 1% methanol containing chloroform. For an hour, the resultant mixture was mechanically swirled at 40 °C. After this, a transparent lipid layer was formed on the flask’s rounded bottom after the mixture was evaporated under lower pressure using a rotary evaporator. The remaining solvents were removed under vacuum for one night. A glycerol–water solution (20% *w*/*v* glycerol) was used to hydrate the thin glycerosomal film. After that, the mixture was mechanically agitated for an hour at 40 °C. Subsequently, the vesicles underwent ultra-sonication. The generated glycerosomes were lyophilized after centrifuging at 4 °C at 7500 rpm to eliminate any extra unentrapped drug [54]. In addition, glycerosomes were prepared by a reverse phase evaporation approach. The lipid mixture was added to the glycerin–water solution, and then the two phases were added to the volatile organic solvent. A stable vesicle was formed by evaporating the solvent. As a contrast to the thin film technique, this approach enables a more effective encapsulation of hydrophilic medicines [51].

Glycethosomes were also prepared by the hot method, as described by Pleguezuelos-Villa et al. [63] and Abdallah et al. [59]. The lipid phase, including phospholipid and other lipid materials, was dissolved in ethanol at elevated temperatures, at around 40–60 °C for better dispersion. Glycerol was then added to the prepared solution under constant stirring. The aqueous phase, including water, was heated at the same temperature as the ethanol–phospholipid solution. The aqueous phase was added gradually to the lipid phase while maintaining the temperature. The stirring process continued until the vesicles were formed.

Another method for producing glycethosome vesicles is the ethanol injection and sonication method, which is described by Zhang et al. [26]. In a glass vial, the drug and lipid combination were first weighed and then dissolved in ethanol. The aqueous phase consisted of a glycerol and water combination. The ethanol phase was introduced into the aqueous phase until glycethosomal dispersion formed. The resultant dispersion is then sonicated, which is typically performed for a few minutes using a probe sonicator, to guarantee homogeneity and reduce the size of the glycethosomes.

#### 3.3.2. Formulation of Phospholipid Nanovesicular Gel

A vesicular gel is a semisolid drug delivery system consisting of a dense network of phospholipid vesicles dispersed in an aqueous phase. This structure allows for prolonged and controlled drug release, making it suitable for small molecule drugs, peptides, and proteins. Phospholipid vesicular gels are valued for their high encapsulation efficiency, biocompatibility, and depot formulation capabilities, which enhance drug stability and therapeutic efficacy [64].

The primary preparation techniques for phospholipid vesicular gel systems include high-pressure homogenization, dual centrifugation, and magnetic stirring. High-pressure homogenization involves subjecting a lipid–drug mixture to high shear forces, resulting in homogeneous gels with high encapsulation efficiency [65]. Dual centrifugation on the other hand employs counter-rotating movements to facilitate rapid mixing, offering advantages such as reduced contamination risk and compatibility with aseptic manufacturing [66]. This dual motion generates high shear forces that effectively homogenize viscous lipid and aqueous phases, leading to uniform vesicle formation. This technique offers several advantages such as solvent-free processing, rapid and efficient mixing, scalability, and sterile production; unlike high-pressure homogenization, dual centrifugation applies lower mechanical stress, making it ideal for proteins and peptides [67]. Magnetic stirring is one of the simplest and most widely used methods for preparing vesicular phospholipid gel systems. This method involves mechanical agitation of phospholipids and an aqueous drug solution using a magnetic stirrer and a stir bar [68]. In the magnetic stirring method for vesicular phospholipid gel preparation, dry phospholipids are accurately weighed and added to an aqueous drug solution or buffer. The mixture is continuously stirred using a magnetic stirrer at room temperature or slightly elevated temperatures for 30 to 120 min, allowing the lipids to hydrate and form vesicles. The agitation promotes the formation of a semisolid, homogenous gel composed of closely packed vesicles and lamellar structures. Moreover, vesicular gels can also be prepared through the incorporation of gelation agents into vesicular systems. These systems have found applications in drug delivery due to their ability to improve the stability, permeation, and controlled release of active substances [69,70].

Encapsulation techniques for drug loading in phospholipid vesicular gels vary based on the nature of the active ingredient. Hydrophilic drugs are often directly incorporated during gel preparation (direct loading), while lipophilic and amphiphilic compounds are integrated into the lipid phase before hydration. Passive loading, where drugs diffuse into pre-formed phospholipid vesicular gels, has also been explored, although it is less efficient for certain compounds [64].

In order to achieve the appropriate consistency, stability, and controlled release characteristics, phospholipid vesicular gels are frequently made by adding different gelling agents. carbopol, a polyacrylic acid derivative that stabilizes the vesicle dispersion and enhances viscosity, is one of the most-used gelling agents [71]. Cellulose derivatives called hydroxypropyl methylcellulose (HPMC) and sodium carboxy methylcellulose (Na CMC) are frequently utilized because of their capacity to create films and deliver regulated medication release [69,72]. Thermoreversible block copolymers called poloxamers (Pluronic) produce gels that change from a liquid to gel in response to temperature variations, which makes them perfect for applications requiring controlled release [73]. Another natural polysaccharide, pectin, is appropriate for topical drug delivery systems because it gels when acid and divalent ions are present. A synthetic polymer called polyvinyl alcohol (PVA) creates elastic, stable sheets that improve the vesicular gel’s stability and controlled release [74]. Last but not least, chitosan, a biopolymer made from chitin, is employed because it enhances the bioavailability of active ingredients and can gel in acidic conditions [75].

### 3.4. Mechanism of Actions of Phospholipid Nanovesicular Systems for Enhanced Transdermal Delivery of Drugs

Novel techniques for delivery that improve skin penetration using phospholipid soft vesicles were created about 40 years ago. The inclusion of compounds like ethanol and glycols gives these phospholipid vesicles their distinctive fluid lipid bilayers. The lipid bilayers in the stratum corneum are fluidized and broken by the solvents in these carriers when applied topically, allowing soft vesicles to penetrate deeper into the skin and release their active ingredients [19,20,32,50] (Figure 1). Therefore, there is a growing emphasis on the treatment of dermatological diseases by the topical application of drugs delivered via these novel carriers.

**Figure 4:**
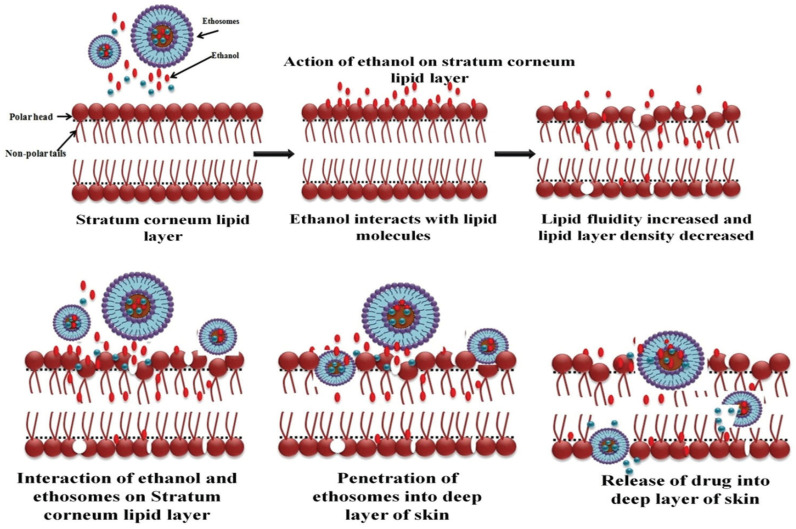
Proposed mechanism of penetration of ethosomes [77].

The Ethosomal Drug Delivery System operates through a unique mechanism of action that enhances the permeation of drugs across biological membranes. This system utilizes ethosomes, which are lipid-based carriers that encapsulate the drug, allowing for improved absorption and targeted delivery. The ethosomes facilitate the transport of the drug through the skin or other barriers, ensuring effective therapeutic outcomes. The interaction between ethosomes and skin lipids is responsible for better drug delivery via ethosomes as opposed to liposomes. The ethanol effect, in which ethanol interacts with the lipid molecules in the polar head group region, is thought to be responsible for the permeation’s initial phase, as seen in Figure 4. Because of this interaction, the lipids in the stratum corneum have a lower transition temperature, which increases their fluidity and lowers the density of the lipid multilayer. This is followed by the “ethosome effect”, which is the release of the drug into the deeper layers of the skin through lipid penetration and permeation through the development of new routes, ascribed to the flexibility and fusing of ethosomes with skin lipids. Vesicles can be made softer and more flexible by ethanol, which makes it easier for them to enter the skin’s deeper layers. The fusing of ethosomes with skin lipids may facilitate drug release along the penetration pathway, resulting in the release of the drug in the deeper layers of the skin and transdermal absorption [76].

In contrast to ethosomes, glycerosomes—vesicular carriers altered by trihydric alcohol—have a distinct mode of action. Glycerol somewhat decreased the Tm of phospholipid bilayers, as was previously mentioned. These vesicles showed at least a 10% rise in their deformability index. Similar to transfersomes, these vesicles’ flexibility and deformability offer a possible pathway for skin penetration. The results of the skin penetration study showed that the amount of glycerol in glycerosomes had a direct impact on their ability to improve drug localization, such as diclofenac, in the epidermal layers [50,62,78]. Additionally, it was suggested that glycerol in the extracellular SC lipids could improve ceramide head group mobility and promote fluidity in the bilayers’ hydrophobic regions [79]. According to a recent study, glycerosomes fluidize the skin’s lipid bilayers, which enhances fisetin penetration. A Tm peak at 45.12 °C, which was present in untreated normal skin, was absent from rat skin treated with glycerosomes carrying the drug, according to a differential scanning calorimetry analysis. The authors explained that the disintegration, reorientation, and increased fluidity of stratum corneum lipids caused the structural changes in the skin. It was proposed that glycerol in glycerosomes improved the fluidity of the lipid bilayer by enhancing the diffusion of the formulation by interacting with the polar head groups of the lipids and facilitating skin hydration [49].

The transdermal flow of paeoniflorin from glycerosomes was significantly better than that of liposomes and a tincture of paeoniflorin, according to in vitro studies [80]. Liposomes have been reported to be restricted to the stratum corneum’s outer layers and incapable of penetrating deeper into the skin [81]. On the other hand, because of their better deformability, ethosomes may increase the mobility of skin lipids, enhancing the absorption of drugs into the stratum corneum [82]. The effectiveness of percutaneous absorption is greatly impacted by the presence of short-chain alcohols in ethosomes. The dense arrangement of lipid molecules may be altered by the addition of short-chain alcohols to ethosomes, which may lower the phase transition temperature of the lipid bilayer in the stratum corneum and cause phase separation and the change of solid and liquid lipids [83]. Glycerol can increase the flexibility and fluidity of the liposomal bilayer and increase the affinity of substances for the aqueous dermal layers because of its high viscosity and hygroscopic qualities. This would increase the ability of glycerosomes to pass through the skin barrier [50].

**Figure 5:**
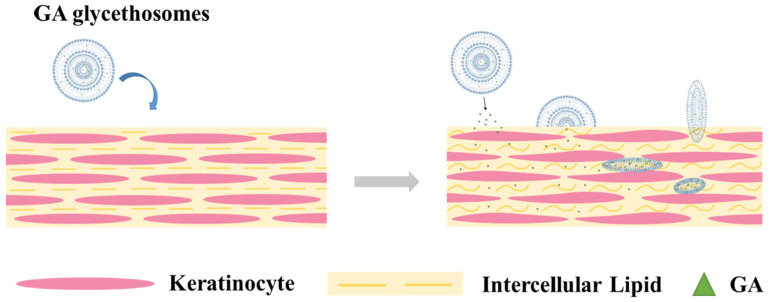
An illustration of how glycyrrhetinic acid (GA) glycethosomes affect the lipids in the stratum corneum [26].

Glycethosomes effectively integrate the benefits of glycerosomes and ethosomes. The lipid extraction was effective, greatly improving lipid fluidity, which may account for the optimal transdermal impact. The mechanism of action is illustrated in Figure 5. According to earlier research, liposome vesicles function as a skin drug delivery system that improves drug penetration via a variety of mechanisms, such as indirect skin penetration, vehicle absorption and/or fusion with the stratum corneum, and the free drug mechanism [84]. Additionally, glycethosomes can reduce skin barrier resistance and greatly increase lipid fluidity in the stratum corneum, which promotes transdermal permeability [26].

### 3.5. Application of Phospholipid Nanovesicular Gel Systems Containing Glycerol and/or Ethanol for Transdermal Drug Administration

As shown in Table 1, this review will look at the details of novel nanovesicular carriers for the skin delivery of pharmacological agents.

**Figure 6:**
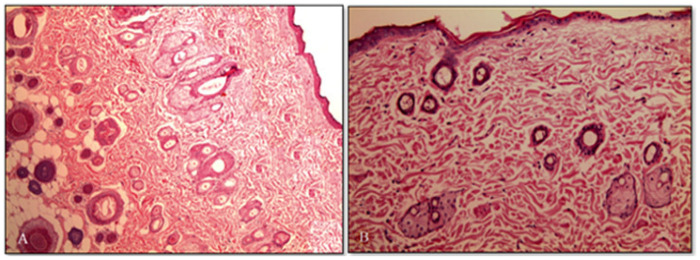
Photomicrograph of section of (**A**) untreated rat skin (control) and (**B**) treated rat skin with optimized ethosomal gel formulation (K-EGF) [85].

Ansari et al. formulated and described karanjin-loaded ethosomal gelling system for improved topical administration and successful treatment of skin acne [85]. Karanjin-loaded ethosomal vesicles demonstrated increased penetration with a 1.9-fold increase in flux and a 2.4-fold greater skin deposition relative to the karanjin hydro-ethanolic solution. The formulated ethosomes were included in the carbopol gel for optimal application on the skin surface. The ethosomal gel (K-EGF) demonstrated enhanced penetration in rat skin, as shown by CLSM, which was used to determine the penetration depth of the ethosomal gel formulation (K-EGF) compared to a hydro-alcoholic solution of rhodamine B [85]. These results can be attributed to the synergistic “ethosomes effect“ and “ethanol effect”, which facilitate the distribution of rhodamine B through ethosomal gel formulation [100]. The Draize scoring test and histological examination showed that the ideal K-EGF formulation did not cause skin irritation. The number and size of sebaceous gland units in the dermis significantly decreased, according to histopathologic analysis, suggesting that ethosomal treatment has an anti-acne effect (Figure 6). Carrageenan-induced edema in the rat paw shown notable anti-inflammatory effects; K-EGF and a traditional anti-inflammatory drug inhibited edema by 66.66% and 70.37%, respectively. The number and size of sebaceous gland units in the dermis were considerably decreased by the ethosomal gel (K-EGF) treatment, which showed anti-acne effects. The results suggest that ethosomal gel, as an efficient carrier system, may be used to improve the topical delivery of karanjin in acne treatment.

Ajikumar et al. concluded that the ethosomal gel of clarithromycin could boost drug bioavailability, reduce administration frequency, improve patient compliance, and maintain sustained drug release [86]. The ethosomal gel was cost-effective and released drugs better than the traditional dosage forms.

Shetty et al. [87] developed ethosomal gel containing clove oil and assessed its efficacy in treating cutaneous candidiasis. Ethosomes of clove oil were produced with varied amounts of Soyaphosphatidylcholine and ethanol, and then subsequently included in carbopol 974 base gels for the formulation of ethosomal gel. The optimized formulation did not induce any skin irritation as the formulation’s pH was within the skin’s pH range. The ethosomal gel demonstrated commendable antifungal efficacy against the fungus C. albicans in comparison to pure clove oil. These findings indicate that the formulated product could be a potential option for the topical administration of clove oil in the treatment of cutaneous candidiasis. The ethosomal gel formulation has demonstrated efficacy by enhancing drug penetration through the skin, hence decreasing dosage, limiting the frequency of administration, and preventing unwanted effects compared to creams, solutions, or liposomes. The results indicated that this formulation may be a potential option for the topical application of clove oil in the treatment of fungal infections.

Eberconazole nitrate (EBZ)-loaded ethosomes were produced by Gupta et al. [88]. After being included into the hydrogel matrix (carbopol^®^ 940), the optimized batch was analyzed for a number of physicochemical properties. The ethosome-loaded hydrogel demonstrated controlled EBZ release in addition to improved skin permeability and EBZ retention when compared to the free drug-loaded hydrogel. The manufactured product’s antifungal efficacy was assessed utilizing an in vivo animal model. According to in vivo tests, ethosomal gel coated with EBZ may lessen fungal infections brought on by a resistant strain of *Candida albicans*. The study showed promising results, suggesting that the pharmaceutical formulation was suitable for treating fungal infections.

Huanbutta et al. created ethosomal formulations containing *Zingiber zerumbet* (L.) Smith. rhizome extract to improve antifungal efficacy in the deep skin layers [101]. The authors utilized broth dilution to ascertain the minimum inhibition concentration (MIC) of ethosomes containing *Z. zerumbet* (L.) rhizome extract against *Candida albicans*. The minimum inhibitory concentration (MIC) of ethosomes containing *Z. zerumbet* (L.) rhizome extract was 312.5 µg/mL, which is five folds greater than the previously reported MIC of *Z. zerumbet* (L.) rhizome extract [102].

Lin et al. developed luridazole ethosomes as an effective transdermal antifungal drug delivery system for efficient drug penetration via the stratum corneum into the skin’s deeper layers. The authors concluded that the developed ethosomes exhibited excellent deformability and stability. The cumulative permeations of various dosage forms after 48 h, ranked from lowest to highest, were as follows: hydro-alcoholic solution > liposomes > ointment > ethosomes. The levels of retention of luliconazole in the skin after 48 h, ranked from lowest to highest, were as follows: hydro-alcoholic solution > liposomes > ointment/ethosomes [90].

Mehmood et al. developed and characterized vitamin D3 gel ethosomes with enhanced anti-psoriasis properties [91]. The drug encapsulation efficiency of ethosomes was 96.25% ± 0.3. The optimized ethosomes had particle diameters of 148 and 657 nm and a PDI of 0.770 ± 0.12. FT-IR, DSC, and TGA investigations showed no interactions between vitamin D3 and other compounds. In total, 95.34% ± 3 of the medicine permeated the membrane. The optimized ethosomal formulation showing the highest entrapment efficiency was incorporated into carbopol 934 gel. Compared to pure vitamin D3, vitamin D3’s large particle size hindered its penetration. Its improvement comes from reduced particle sizes during ED3-7 precipitation. Ethosomes are ethanol-loaded lipid vesicles. Phospholipids, water, and ethanol are typical. Ethosomes are more flexible and may cross the epidermal barrier more easily than ordinary liposomes due to their increased ethanol content. Ethanol breaks the stratum corneum lipid bilayer, making skin more permeable. This disturbance helps drug distribution via the skin.

Al-Ameri et al. developed a meloxicam ethosome hydrogel for topical administration to increase solubility, skin penetration, and effectiveness while avoiding systemic side effects [92]. MLX–Ethos–OF hyaluronic acid hydrogel was made and tested. MLX–Ethos–OF showed efficient permeation with a flux of 70.45 μg/cm^2^/h. Ethosomes are flexible and deteriorates because stratum corneum ethanol and propylene glycol solubilize the skin lipid layer and supply MLX.

Halagali et al. prepared ethosomal formulations loaded with quercetin using a hot technique [93]. Subsequently, quercetin-loaded ethosomal gel and quercetin hydrogel were formulated using 1% carbopol 940 as a gelling agent. In comparison to conventional hydrogel, the findings indicated that ethosomal gel might serve as an effective quercetin transdermal delivery route in the management of inflammation.

Another study by Abdallah et al. designed, optimized, and assessed brucine ethosomal gel’s anti-inflammatory properties [29]. The optimum formulation was mixed with hydroxy propylmethylcellulose gel to develop brucine ethosomal gel. The obtained results demonstrated that ethosomal gel extended drug release in vitro for 6 h. The permeability of the drug from brucine ethosomal was substantially greater than brucine gel, and brucine suspension was *p*  <  0.05. Subsequently, after 24 h, BRU ethosomal gel significantly reduced inflammation in the hind paws of rats, indicating its strong anti-inflammatory effects. The study concluded that ethosomal gel could enhance brucine’s anti-inflammatory efficiency.

Another study by Valsalan et al. aimed to increase alpha phellandrene’s skin compatibility and permeability using an ethosomes gel for gout therapy, since oral administration causes gastrointestinal problems and toxicities [94]. Drug-loaded ethosomes were loaded into a carbopol gel to prepare ethosomal gel (APEG). The anti-inflammatory effectiveness of the generated formulation was measured by its inhibitory effects on cellular nitrite in RAW 264.7 cells, inducible nitric oxide synthase (INOS), lipoxygenase-5 (LOX-5), cyclooxygenase II (COX-2), and myeloperoxidase (MPO). The significant enzyme inhibition indicated the anti-inflammatory effectiveness of the formulations. The transdermal ethosomal formulation of alpha phellandrene is an effective alternative to oral usage.

A promising approach to improve the transdermal delivery of poorly soluble bioactive compounds, such as piperine (PE), involves the use of binary ethosomes (ES). In a recent study by Zafar et al., PE-loaded ethosomes gel (PEES-gel) was developed to enhance the bioavailability and therapeutic efficacy of PE [103]. The ethosomes were prepared using the hot method and optimized through experimental design software, with key parameters such as phospholipid, cholesterol, and alcohol mixture composition being evaluated for their impact on vesicle size and entrapment efficiency. The optimized PE-loaded ethosomes (PEESopt) exhibited favorable characteristics, including a vesicle size of 187.7 nm, a PDI of 0.253, and an entrapment efficiency of 75.12%. When incorporated into carbopol and HPMC-K100 gels, the PEES-gel demonstrated excellent viscosity, spreadability, and biocompatibility with skin pH. The gel showed significantly higher and sustained drug release (86.81% in 24 h) compared to PE gel (51.20%) and enhanced skin permeation, with a 1.99-fold increase in flux. Furthermore, the PEES-gel exhibited superior antimicrobial and antioxidant activities compared to pure PE, suggesting that it can serve as an effective and natural alternative for the topical delivery of PE.

A study by Yasmin et al. aimed to develop glycerosomes loaded with tolnaftate using 30% *v*/*v* glycerol as distinct carriers for cutaneous (trans) drug delivery systems [95]. The obtained glycerosomes were evaluated using liposomes as control. The ex vivo skin penetration experiments revealed an enhanced drug release compared to traditional liposomes and solution. Skin irritancy trials on guinea pigs demonstrated no toxicity. In conclusion, glycerosomes are a promising transdermal drug delivery technology for tolnaftate.

A study by Shaanya et al. aimed to prepare a vesicular carrier of terbinafine hydrochloride for transdermal administration to treat mycoses [96]. Drugs in vitro/ex vivo release showed better drug penetration than the commercial formulation. Histopathology and cytotoxicity showed that the glycerosomal carrier system could distribute terbinafine hydrochloride transdermally. Terbinafine hydrochloride glycerosomes may be used to formulate skin infection treatments.

A study conducted by Alam et al. attempted to create carbopol gel-encapsulated glycerosomes for sunburn treatment [56]. The optimum glycerosomal formulae was integrated into carbopol 934P gel. According to a dermatokinetics study, glycerosomal gel showed better penetration than conventional gel. Based on a skin irritation investigation, the formulation has been found to be safe for topical administration. The results show that, when kept under typical conditions, the prepared glycerosomal gel showed stability over extended periods of time.

Zhang et al. conducted a study to enhance the transdermal distribution and synovial absorption of paeoniflorin (PF) through the formulation of glycerosomes carriers with essential oils for topical application [80]. The results showed that the transdermal flow of paeoniflorin encapsulated in STO-glycerosomes was superior to that of similar drug tincture and liposomes in vitro. Significant skin permeability and enhanced drug absorption in the synovium were demonstrated by STO-mediated glycerosomes, suggesting that STO-glycerosomes could be a useful transdermal delivery method for paeoniflorin in the management of rheumatoid arthritis linked to synovial lesions.

Another study conducted be Salem et al. developed glycerosomes vesicles for the administration of celecoxib (CLX) and cupferron (CUP) via skin [97]. This research generated new topical soft vesicles containing CLX or CUP to enhance effectiveness and mitigate systemic toxicity associated with CLX and CUP. The optimized glycerosomes were incorporated into carbopol 934P to develop glycerosomal gel formulations. The application of CLX- and CUP-loaded glycerosomal gel to the skin led to a significant decrease in edema, congested capillaries, and inflammatory cells, suggesting its potential use in treating various inflammatory illnesses.

**Figure 7:**
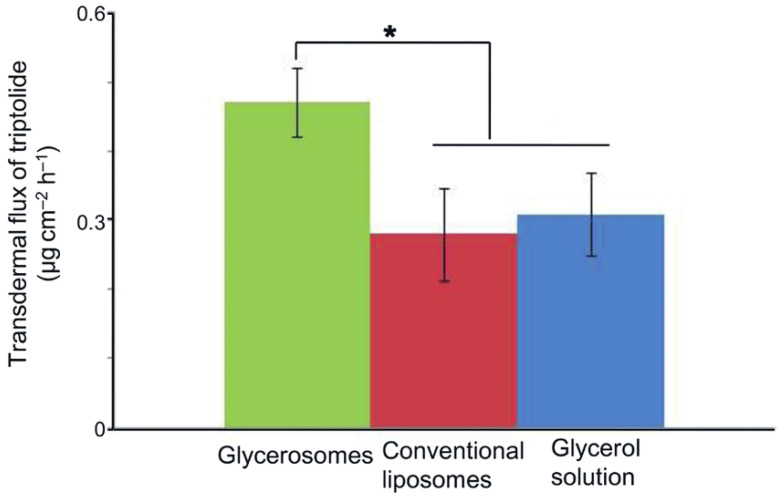
Triptolide transdermal flux in glycerosomal vesicles, conventional liposomal vesicles and 20% glycerol solution (* *p* < 0.05; *n* = 5) [98].

The study by Zhu et al. aimed to optimize the formulation of glycerosomes as drug carriers applied transdermally using triptolide, which has potent anti-inflammatory and immune-modulating properties [98]. Glycerol is used as edge activator to increase liposome deformability and transdermal permeability. The study aimed to optimize glycerosome compositions for the better transdermal distribution of triptolide utilizing an orthogonal experimental approach. The prepared glycerosomes were compared to traditional liposomes regarding an ex vivo skin permeation investigation. In comparison to standard liposomal vesicles and 20% glycerol solution, optimized glycerosomes exhibited 1.78 and 1.52 times the transdermal penetration of triptolide; see Figure 7. Glycerosomes had smaller particle size and greater surface charge than normal liposomes due to their high glycerol content, which improved phospholipid membrane flexibility. Glycerol increased glycerosome vesicle deformability and stratum corneum hydration, boosting triptolide transdermal administration. This study showed that glycerosomes could effectively deliver drugs transdermally, reducing triptolide adverse effects compared to oral treatment.

In a recent study by Singh et al., glycerosomes emerged as an effective delivery system to enhance the skin permeation of poorly soluble compounds like Crisaborole (CB), a topical PDE4 inhibitor approved for the treatment of Atopic Dermatitis [104]. A glycerosomal gel formulation of CB was developed using the thin film hydration method, incorporating soya phosphatidylcholine, cholesterol, and varying concentrations of glycerol. The optimized glycerosomes demonstrated a vesicle size of 137.5 ± 50.58 nm, a PDI of 0.342, and a zeta potential of −65.4 ± 6.75 mV, with an entrapment efficiency suitable for topical application. The glycerosomal gel exhibited a 2.13-fold enhancement in permeation flux compared to the conventional 2% CB ointment. This study highlights the potential of glycerosomes as an effective delivery system for enhancing the transdermal penetration of poorly soluble drugs like CB.

Another study by Alhakamy et al. developed, optimized, and compared plumbagin-loaded glycerosomal gel to traditional liposomal vesicles for skin cancer treatment [99]. Because of its poor water solubility and bioavailability, plumbagin (PLM) has limited clinical utility in the destruction of cancer cells. A pre-formed gel was made from the optimum PLM-loaded glycerosomes and carbopol 934 polymer. The diffusion fluxes of glycerosomal-loaded gel, conventional liposomal gel, and drug suspension were 79.43 ± 12.43, 23.31 ± 6.0, and 12.3 ± 4.5 µg/cm^2^/h, respectively. The significant permeation observed from the glycerosomes gel results from the elastic and ultra-deformable features of the vesicle. This characteristic facilitates the drug’s deeper penetration into the skin layers. Furthermore, high-performance liquid chromatography (HPLC) was used to measure the PLM concentration in the stratum corneum, epidermis, and dermis. A considerable quantity of PLM was deposited in the epidermis and dermis from GM-loaded gels as opposed to drug suspensions and traditional liposomal gels. This is likely attributed to the elastic and ultra-deformable properties of glycerosomes in addition to the edge-activating effect. The confocal images showed that glycerosomal gel penetrates the skin deeper than the control solution. The glycerol modified the lipid bilayer’s fluidity within the vesicle and entered the skin’s deeper layers through tiny pores [105,106].

The study by Pleguezuelos-Villa et al. aimed to develop a novel treatment for skin illnesses such as psoriasis and atopic dermatitis [63]. Glycethosomes were employed to transport mangiferin, an anti-inflammatory and antioxidant substance, to the skin. The mangiferin skin penetration and retention in human skin samples were also evaluated. The study tested mangiferin-loaded glycethosomes in an inflammatory mouse skin disease model for therapeutic effectiveness. In vitro investigations on human abdomen skin showed that vesicles enhance mangiferin retention in epidermis dose-dependently. Adding glycerol and ethanol to the vesicles potentially improved skin penetration and retention. Also, glycethosomes were biocompatible and effectively protected fibroblasts from hydrogen peroxide injury in vitro. The in vivo data showed that mangiferin-loaded glycethosomes promoted wound healing better than dispersion, suggesting their potential use for TPA-induced wounds. This study shows that glycethosomes vesicles can increase the distribution and efficacy of antioxidant substances like mangiferin for inflammatory skin disorders.

A study by Zhang et al. developed a novel liposome system using glycerol and ethanol to stabilize vesicles and increase glycyrrhetinic acid (GA) skin penetration [26]. Ethanol injection and sonication was utilized to produce glycethosomes. HPLC was used to measure GA content in various skin layers of pig skin in Franz cells during in vitro permeation testing. Glycethosomes exhibited the best transdermal impact and had a 20.67% skin penetration rate, whereas drug dispersion, liposomes, glycerosomes, and ethosomes had rates of 5.02%, 7.78%, 9.38%, and 10.56%, respectively. Studies suggest that adding glycerol could enhance the transdermal impact. Glycerol hydrates the lamellar matrix, allowing vesicles to reach deep skin layers [107]. Many investigations have demonstrated that ethanol enhances transdermal permeation, allowing medicines with limited permeability to permeate deeper into the skin layers and improve drug efficiency [108]. The glycethosomes successfully extracted and fluidized skin stratum corneum lipids. This study revealed that using glycethosomes greatly enhanced the transdermal action of GA. Glycethosomes possess an apparent capacity to extract lipids and enhance the fluidity of those lipids, which results in a skin GA content that is more than double that of other vesicles. Experiments indicate that glycyrrhetinic acid incorporated in glycethosomes shows promise for use in cosmetics and for treating skin problems.

## 4. Conclusions

The developments in nanovesicular systems for the enhanced skin delivery of medicinal compounds are highlighted in this study. These include a wide range of research that concentrates on their development from the traditional liposomes to more advanced vesicular systems including ethanol and/or glycerol. This paper draws attention to the noteworthy developments achieved by creatively altering the composition and characteristics of vesicles in order to bypass the skin barrier. The prospective function of these kinds of carriers is demonstrated by various in vitro, in vivo, and clinical studies on the transdermal distribution of a broad range of medicinal compounds. Active compounds integrated into nanovesicles have proven effective in treating systemic illnesses and skin conditions. This evaluation will direct and assist researchers in selecting an appropriate carrier for the creation of novel transdermal systems. Despite the numerous advantages of transdermal drug delivery systems (TDDS), issues such drug stability, restricted skin permeability, and accurate dosage management continue to be major obstacles. To improve medication absorption and targeting, future studies should concentrate on advanced formulation techniques including microneedles, nanoparticles, and stimuli-responsive devices. Further advancements in these fields might increase the clinical usefulness of TDDS, enhance therapeutic results, and provide patients with more convenient, efficient treatment choices.

The potential of modified phospholipid vesicular gels as advanced transdermal drug delivery vehicles that provide enhanced drug penetration, stability, and therapeutic effectiveness is highlighted in this review. We provide researchers and formulators with a useful resource for choosing and refining these systems for upcoming pharmaceutical applications by highlighting important formulation techniques, processes, and current developments. To further improve these delivery systems, future research should concentrate on investigating advanced modifications and innovative applications. To improve accuracy and effectiveness, this involves creating stimuli-responsive carriers, using site-specific targeting techniques, and integrating smart technologies like nanotechnology. These developments might help these systems overcome current drawbacks and increase their therapeutic potential in a wider range of clinical application.

## Figures and Tables

**Table 1:** Phospholipid vesicular gel systems for effective transdermal delivery of active molecules.

Active Molecules	Indication	Composition	Method of Preparation	Key Findings	References
Karanjin	Acne vulgaris	Phospholipids 90 G (30 mg, *w*/*w*) and drug karanjin (3% *w*/*v*) were dissolved in chloroform–methanol (2:1, *v*/*v*)/isotonic phosphate buffer–ethanol solution (25:75) was used as hydrating medium	Film hydration method	The produced ethosomal gel has physicochemical features suitable for dermal medicinal drug delivery; the ethomal gel showed the significant antibacterial and antioxidant properties of karanjin.	[85]
Clarithromycin	Acne vulgaris	Phospholipid, ethanol, propylene glycol, drug, and distilled water	Cold method	The study concluded that the ethosomal gel of clarithromycin could effectively improve the bioavailability of the drug by penetration enhancement, reduce the frequency of administration, give better patient compliance and also follow a sustained drug release mechanism.	[86]
Clove oil	Cutaneous candidiasis	Soyaphosphotidyl choline 1–4%, 1–50% ethanol, 0.24 mL of the drug, and aqueous phase up to 100% *w*/*w*	Cold method	The ethosomal gel increased drug penetration through skin, thereby reducing the dose, minimizing frequent application, and preventing adverse effects.	[87]
Eberconazole nitrate	Antifungal	Phosphatidylcholine (PC), ethanol, and propylene glycol (PG)	Cold method	The ethosome-loaded hydrogel demonstrated controlled drug release with higher a skin permeation and skin retention of the drug.	[88]
*Zingiber zerumbet* (L.) Smith. rhizome extract	Antifungal skin infection	1% (*w*/*v*) phosphatidylcholine and 40% (*v*/*v*) ethanol	Cold method	The ethosome system significantly enhanced the skin penetration and retention of the active compound.	[89]
Luridazole	Antifungal	5% (*w*/*v*) lecithin, 45% (*v*/*v*) ethanol	Thin-film hydration	Ethosomal formulation had minimal skin irritation, a better permeation effect, and antifungal activity.	[90]
Vitamin D3	Psoriasis	Soya lecithin 1–8% (*w*/*v*), propylene glycol, and ethanol	Cold process was used with a little bit of modification	Ethosomes loaded with vitamin D3 were successfully prepared and then converted into gel for patients’ easy application. The generated formulation containing vitamin D3 could be useful in overcoming psoriasis.	[91]
Meloxicam	Arthirities	Soya lecithin, ethanol, and propylene glycol	Hot technique	Meloxicam was successfully formulated as ethosomal hydrogel for topical delivery, introducing a promising approach that increases drug skin permeability and efficacy over the classical type.	[92]
Quercetin	Anti-inflammatory and anti-allergy properties	Phospholipid (soya lecithin), cholesterol, and ethanol (20% to 40%)	Hot technique	The developed ethosomal gel could function as an efficient transdermal delivery system for quercetin in the management of inflammation.	[93]
Brucine	Anti-inflammatory effect	Lecithin, cholesterol, and ethanol	Thin film hydration method	The developing ethosomes were successfully used, offering a promising approach for the transdermal delivery of brucine.	[29]
Alpha Phellandrene	Gout	Soya lecithin, cholesterol, ethanol, and propylene	Cold approach	The enhanced skin penetration with deposition indirectly showed the formulation’s topical efficacy.	[94]
Tolnaftate	Antifungal	Hydrogenated soy phosphatidyl choline (HSPC), dipalmitoylphosphatidyl choline (DPPC), distearoylphosphatidyl choline (DSPC), cholesterol, glycerol	Thin film hydration method	Glycerosomes confirmed the effective ability to improve the topical delivery of tolnaftate. The diffusibility of tolnaftate through glycerosomes was appreciable when compared to control liposomes.	[95]
Terbinafine hydrochloride	Mycoses	Phospholipid, cholesterol, and glycerol–water mixture	Thin film hydration method	Glycerosomes could be a potential formulation approach for treating dermal infection and offer a better alternative to commercially available products.	[96]
Rutin	Inflammation associated with sunburn	Phospholipid 90 G, cholesterol, glycerol, and water	Thin film hydration method	Suitable alternation for topical administration of drug to maximize the therapeutic efficacy of the drugs.	[56]
Paeoniflorin	Rheumatoid arthritis	Lipoid S 80, cholesterol, glycerol, and water	Reverse-phase evaporation method	Superior transdermal flux; safe and applicable vehicle for the treatment of rheumatoid arthritis.	[80]
Celecoxib and Cupferron	Inflammatory illnesses	Soybean phosphatidyl choline, cholesterol, glycerol, Tween 80, and water	Hydration film methods	The study confirmed that the anti-inflammatory effect of topically applied drug-loaded glycerosomal gel formulations can have a profound therapeutic application in topical delivery for the treatment of inflammation.	[97]
Triptolide	Various immune and inflammatory diseases and rheumatoid arthritis	Soy lecithin, cholesterol, glycerol	Injection method	The optimized glycerosome formulation would increase triptolide transdermal permeability compared to liposomes.	[98]
Plumbagin	Skin cancer	Phospholipid 90 G, cholesterol, glycerol–water mixture (30% *w*/*v* glycerol)	Thin film hydration technique	The developed formulation exhibited sustained release and gave excellent flux across various strata of cutaneous layer.	[99]
Gycyrrhetinic acid	Anti-inflammatory and antioxidant	Soy phosphatidylcholine (PC60), cholesterol, ethanol, glycerol and water	Ethanol injection and sonication method	Glycethosomes could effectively enhance the fluidity of lipids in the stratum corneum and reduce the skin barrier resistance so as to achieve the effect of promoting transdermal permeability.	[26]
Risperidone	Schizophrenia	Epikuron 200 phospholipid, ethanol, glycerin, and propylene glycol, cholesterol	Ethanol injection and sonication method	The optimized glycethosome gel boosted RS.	[59]

## Data Availability

The original contributions presented in this study are included in the article. Further inquiries can be directed to the corresponding author.
